# Systematic analysis of the molecular mechanism underlying atherosclerosis using a text mining approach

**DOI:** 10.1186/s40246-016-0075-1

**Published:** 2016-06-02

**Authors:** Dan Xi, Jinzhen Zhao, Wenyan Lai, Zhigang Guo

**Affiliations:** Division of Cardiology, Huiqiao Medical Center, Nanfang Hospital, Southern Medical University, 1838 North Guangzhou Avenue, Guangzhou, 510515 Guangdong People’s Republic of China; Laboratory of Department of Cardiology, Nanfang Hospital, Southern Medical University, Guangzhou, 510515 Guangdong People’s Republic of China

**Keywords:** Atherosclerosis, Pathogenesis, Text mining

## Abstract

**Background:**

Atherosclerosis is one of the common health threats all over the world. It is a complex heritable disease that affects arterial blood vessels. Chronic inflammatory response plays an important role in atherogenesis. There has been little success in fully identifying functionally important genes in the pathogenesis of atherosclerosis.

**Results:**

In the present study, we performed a systematic analysis of atherosclerosis-related genes using text mining. We identified a total of 1312 genes. Gene ontology (GO) analysis revealed that a total of 35 terms exhibited significance (*p* < 0.05) as overrepresented terms, indicating that atherosclerosis invokes many genes with a wide range of different functions. Pathway analysis demonstrated that the most highly enriched pathway is the Toll-like receptor signaling pathway. Finally, through gene network analysis, we prioritized 48 genes using the hub gene method.

**Conclusions:**

Our study provides a valuable resource for the in-depth understanding of the mechanism underlying atherosclerosis.

**Electronic supplementary material:**

The online version of this article (doi:10.1186/s40246-016-0075-1) contains supplementary material, which is available to authorized users.

## Background

Atherosclerosis is a complex heritable disease involving multiple cell types and the interactions of many different molecular pathways [1]. Atherosclerosis is therefore a syndrome affecting arterial blood vessels due to a chronic inflammatory response [2, 3]. Atherosclerosis is at the core of cardiovascular diseases, often leading to myocardial infarctions, stroke, and peripheral vascular diseases.

Recent genome-wide association studies (GWAS), involving hundreds of thousands of individuals, have identified numerous loci contributing to atherosclerotic traits and to risk factors such as blood lipoprotein levels and blood pressure [4]. Plasma lipids are primarily of importance for driving early atherosclerosis development, consistent with the notion that loci identified by GWAS will be more useful for primary prevention and with the experimental finding that atherosclerosis regression in response to LDL lowering is much greater for early lesions than for mature and advanced lesions [5]. The extensive ongoing studies into the molecular mechanisms of the 153 confirmed GWA-defined CAD loci will shed light on this issue, as these mechanisms will likely be traceable to early versus late events in the pathogenesis of atherosclerosis [6]. Despite a large number of genes are identified, there has been little success in fully identifying functionally important genes in the pathogenesis of atherosclerosis.

Recently, the text mining methodology has been implemented, providing a necessary means to retrieve disease-related genes in an automated way [7]. Here, we reported on a systematic analysis of atherosclerosis-related genes using text mining. Our study provides in-depth insights into the molecular mechanisms underlying atherosclerosis.

## Results

### Identification of atherosclerosis-related genes by using text mining

**Fig. 1:**
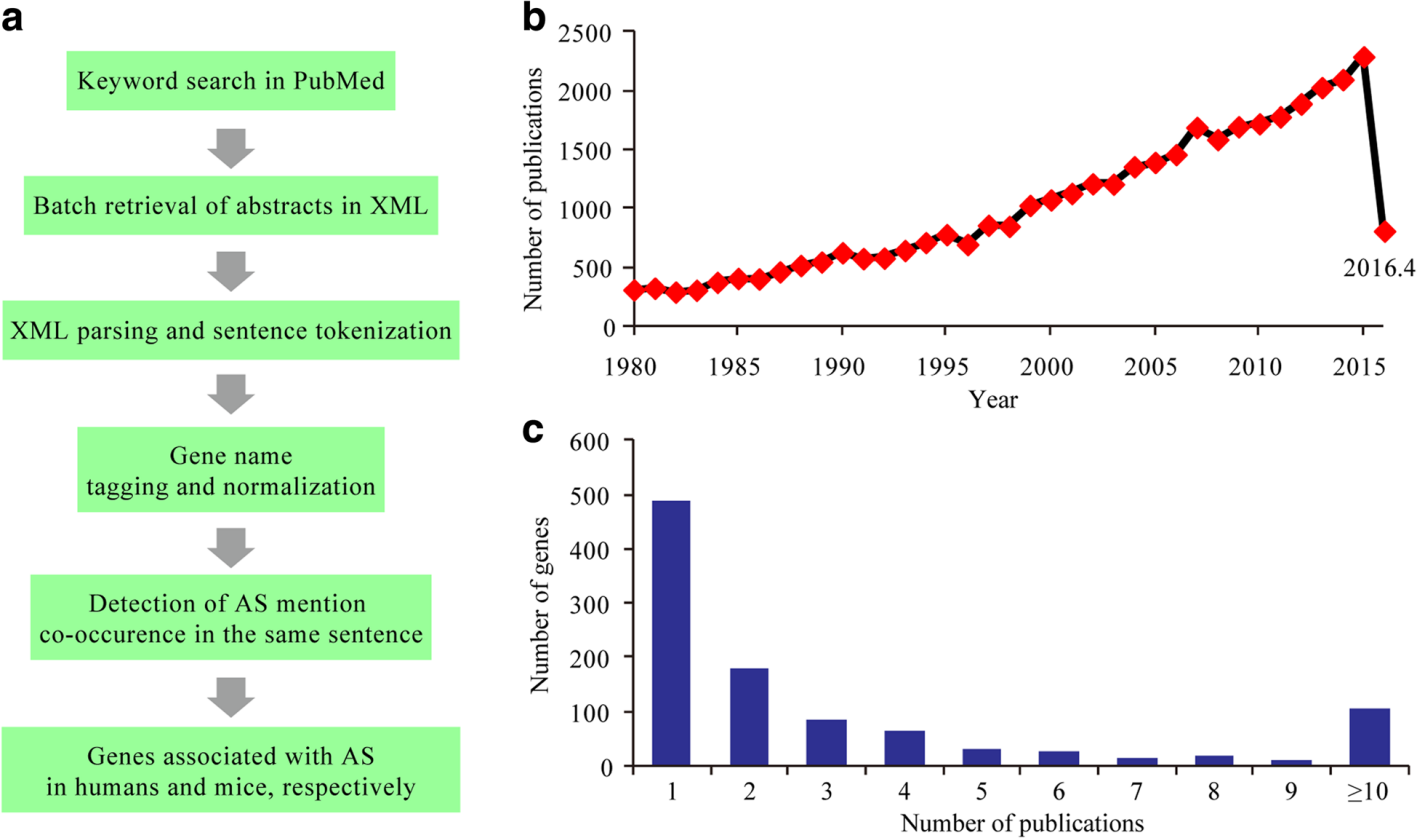
Systematic identification of susceptibility genes for atherosclerosis. **a** Overview of the experimental design. **b** Cumulative number of publications related to atherosclerosis by year (from January 1980 to April 2016). **c** Distribution of the number of publications per gene

We ran a key word search in the PubMed database for articles related to atherosclerosis and obtained 45,304 entries as a result (from January 1980 to April 2016). Abstracts of these articles were downloaded and processed through a text mining pipeline shown in Fig. 1a. Cumulative distribution analysis indicated that the number of articles published on atherosclerosis is growing linearly in recent years (Fig. 1b). From these articles, we extracted atherosclerosis-associated genes via text mining. We compiled a list of 1312 atherosclerosis-related genes (Fig. 1c; Additional file 1: Table S1).

### Functional clustering analysis

**Fig. 2:**
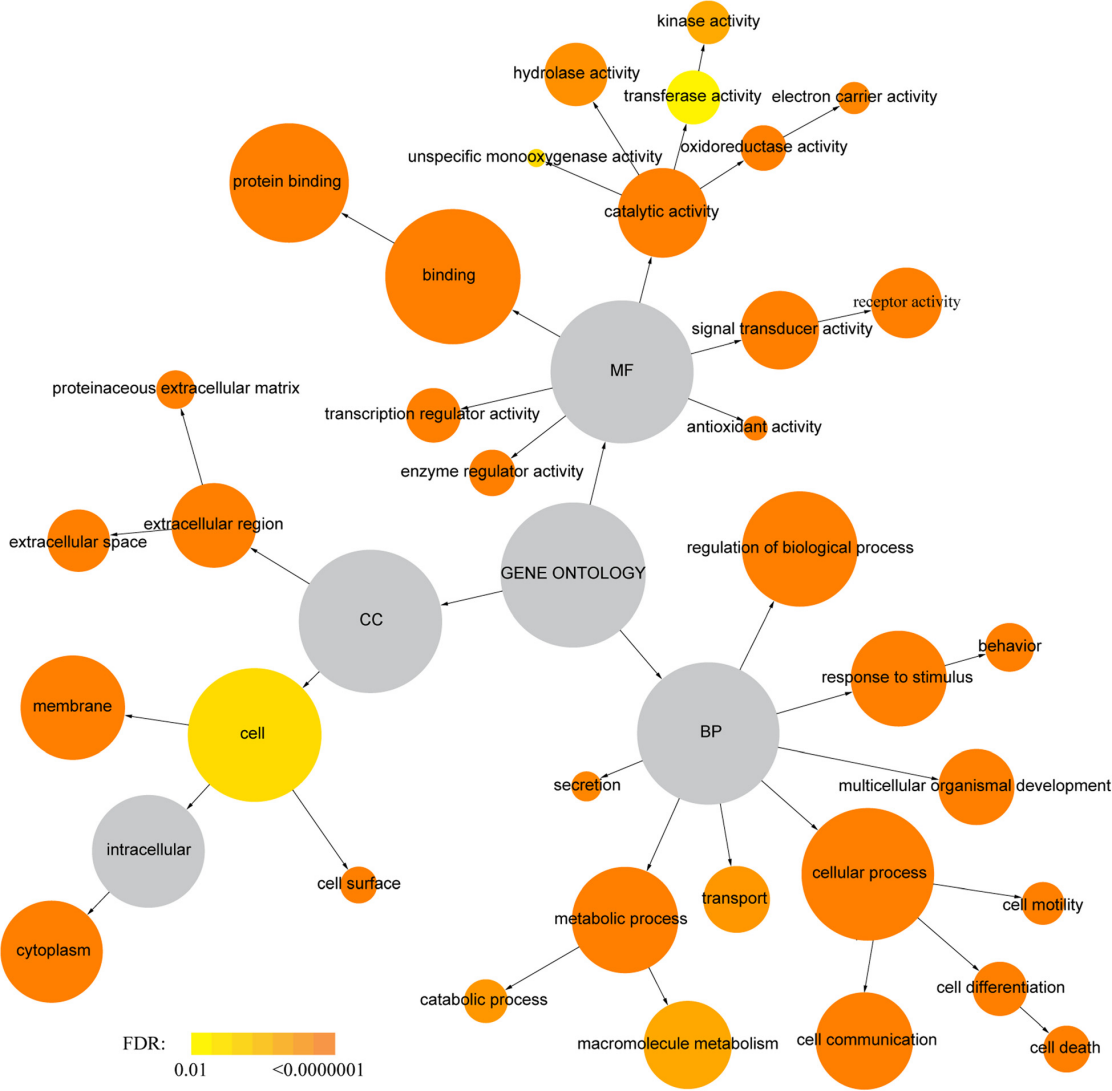
Gene ontology (GO) enrichment analysis of atherosclerosis-related genes. GO analysis was performed by using the BiNGO software. GOslim categories with significant enrichment were highlighted with different colors representing different levels of significance. The sizes of circles are proportional to the number of genes

All 1312 unique genes were functionally categorized based on gene ontology (GO) annotation terms using the BiNGO program package. Enrichment analysis revealed that a total of 35 terms exhibited significance (*p* < 0.05) as overrepresented terms. In the biological process category, response to stimulus, cell communication, regulation of biological process, cellular process, behavior, multicellular organismal development, cell motility, cell death, metabolic process, cell differentiation, enzyme regulator activity, transcription regulator activity, electron carrier activity, secretion, catabolic process, transport, macromolecule metabolic process, and unspecific monooxygenase activity were found to be significantly enriched. GO terms related to extracellular region, extracellular space, cell surface, cytoplasm, membrane, proteinaceous extracellular matrix, and cell were overrepresented under the cellular component category. The overrepresented GO terms in the molecular function category were protein binding, binding, signal transducer activity, receptor activity, antioxidant activity, oxidoreductase activity, catalytic activity, hydrolase activity, kinase activity, and transferase activity (Additional file 2: Table S2). The hierarchical organization of these GO terms is shown in Fig. 2, together with the significance of enrichment indicated by different colors.

### Pathway analysis

**Fig. 3:**
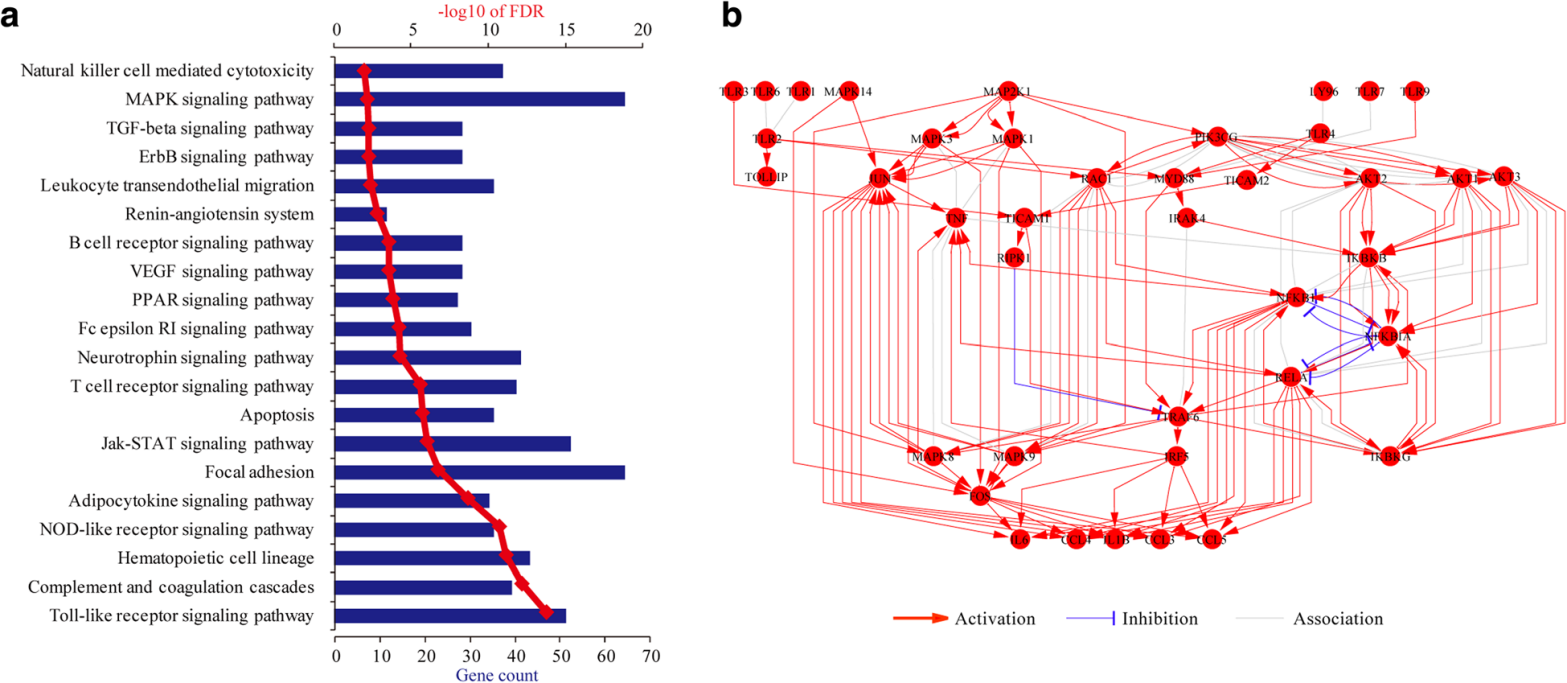
Pathway analysis of atherosclerosis-related genes. **a** Enrichment analysis of pathways. DAVID online tools were used and genes are classified according to the KEGG pathway database. **b** Visualization of the Toll-like receptor signaling pathway. Nodes represent genes. Edges represent gene dependences derived from KEGG pathway hsa04620. Genes without a direct interaction with others are not included. This graph was generated by using the Cytoscape software

In addition to the GO analysis, we also performed pathway analysis by using the DAVID tools. Unlike GO, which only contains lists of functional gene groups, the pathway database also stores the information of gene dependencies in each pathway. In the present study, all atherosclerosis-related genes were linked to a total of 50 pathways. Among these pathways, 20 pathways, namely Toll-like receptor signaling pathway, complement and coagulation cascades, hematopoietic cell lineage, NOD-like receptor signaling pathway, adipocytokine signaling pathway, focal adhesion, Jak-STAT signaling pathway, apoptosis, T cell receptor signaling pathway, neurotrophin signaling pathway, Fc epsilon RI signaling pathway, PPAR signaling pathway, VEGF signaling pathway, B cell receptor signaling pathway, renin-angiotensin system, leukocyte transendothelial migration, ErbB signaling pathway, TGF-beta signaling pathway, MAPK signaling pathway, and natural killer cell mediated cytotoxicity were significantly enriched (*p* < 0.05) (Fig. 3a; Additional file 3: Table S3). Based on enrichment *p* value, the most highly overrepresented pathway went to the Toll-like receptor signaling pathway (Fig. 3b). The Toll-like receptor signaling pathway is known to play an important role during atherosclerosis in both immune and inflammatory response.

### Network analysis

**Fig. 4:**
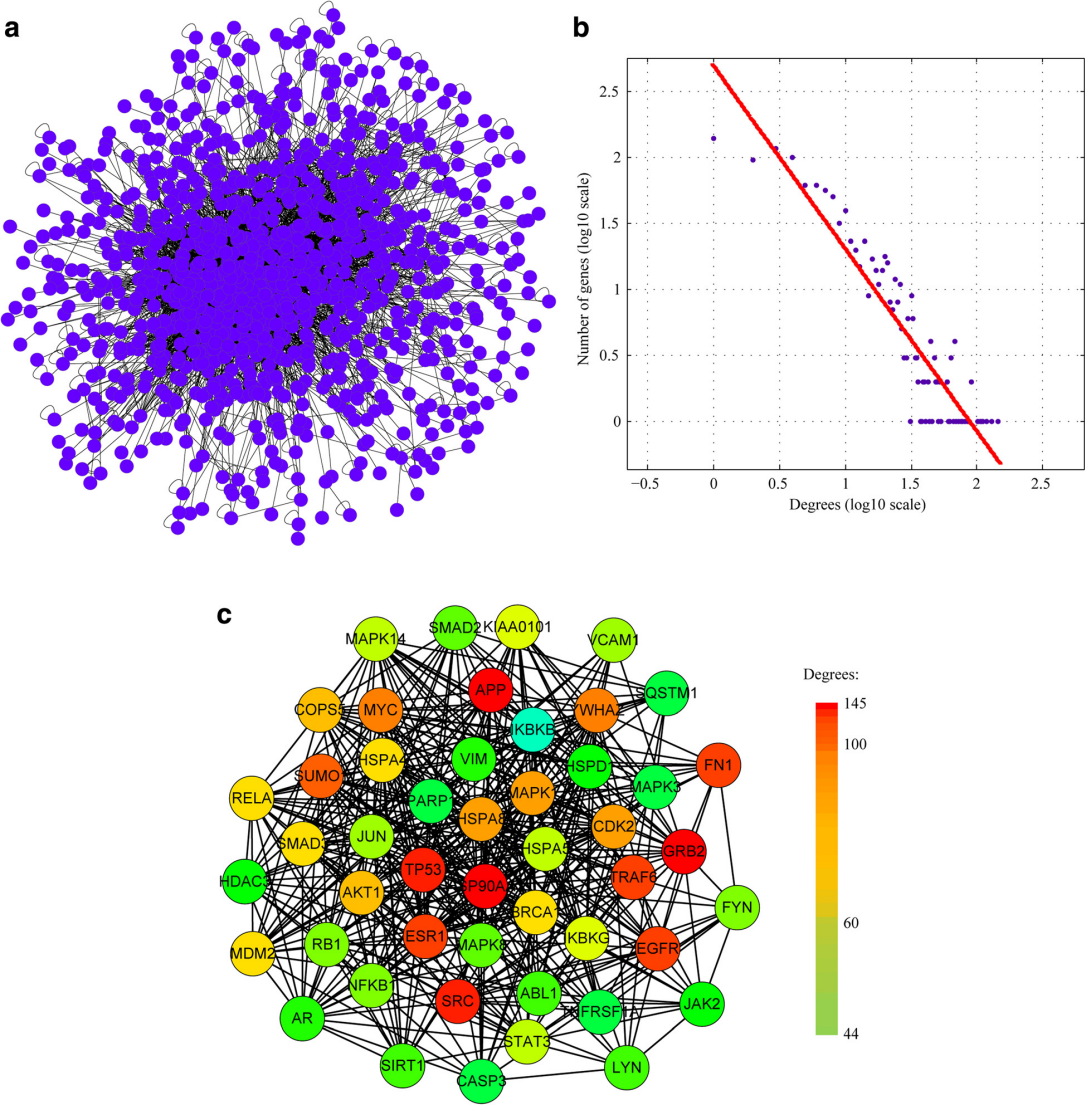
Protein-protein interaction (PPI) network of atherosclerosis-related genes. **a** PPI network of atherosclerosis-related genes. **b** Degree distribution of the PPI network. The degree distribution follows a power law distribution. **c** The simplified PPI network of hub genes

In the present study, a genome-wide protein-protein interaction (PPI) network was constructed by merging up-to-date protein-protein interactions available in IntAct [8], BioGRID [9], MINT [10], DIP [11], HPRD [12, 13], and MIPS [13]. The network related to atherosclerosis was generated by mapping the atherosclerosis-related genes to the genome-wide PPI network. The atherosclerosis network consisted of 1079 nodes connected via 6089 edges (Fig. 4a). Topological analysis showed that the network follows a power-law distribution (Fig. 4b) and therefore is a scale-free, small-world network [14]. This type of networks has the particular feature that some nodes are highly connected compared with others within the network. These highly connected nodes, also known as hub genes, represent important genes in the network and therefore are treated with special attention. Using a defined cut-off value, we identified 48 hub genes. These hub genes and their connections were extracted from the whole network and rendered as a simplified sub-network (Fig. 4c).

## Discussion

In the present study, we attempted to compile a complete list of genes involved in atherosclerosis. In recent years, high-throughput transcriptomic and proteomic approaches make it possible for studying the expression levels of thousands of genes and proteins simultaneously. However, these data suffer from high technical variability and high dimension size [15, 16]. On the contrary, there is a large body of research using conventional gene-by-gene methods. Text mining provides the necessary means to retrieve these data through automated processing of texts [7]. Here, we performed a text mining analysis of atherosclerosis-associated genes. We identified 1312 genes from 45,304 publications. Considering the large body of literature we analyzed, our result may have reasonably good coverage of all atherosclerosis-associated genes.

We found that 1312 genes were associated with atherosclerosis. Based on GO analysis, 35 GO terms were significantly enriched. Additionally, our study also revealed 20 enriched pathways. Based on enrichment *p* value, the most highly overrepresented pathway went to the Toll-like receptor (TLR) signaling pathway. The Toll-like receptor signaling pathway is known to play an important role during atherosclerosis in both immune and inflammatory response. The disruptions of cellular or organismal cholesterol homeostasis that occur as a risk factor of atherosclerosis may lead to an augmentation of inflammatory responses via enhanced TLR signaling or inflammasome activation [17]. TLR activation leads to the expression of pro-inflammatory cytokines and also induces the expression of many negative regulators, acting to limit signal transduction, messenger RNA (mRNA) transcription, or translation [18].

A genome-wide gene network was constructed by using up-to-date interaction data available in the PINA2 database [19]. We obtained a gene network consisting of 1079 nodes connected via 6089 edges. So far, several studies have been conducted to incorporate the topology of gene network in prioritization of disease candidate genes [20–22]. The main concern for these studies is that the incompleteness and noisiness of interaction data may affect the accuracy of prioritization result. By merging up-to-date protein-protein interactions available in IntAct [8], BioGRID [9], MINT [10], DIP [11], HPRD [12], and MIPS [13], the PINA2 database provides a comprehensive gene network at genome-wide scale. We expected that the use of the PINA2 database may alleviate this problem to a certain extent. Using a defined threshold value for degree, we identified a total of 48 hub genes in this network.

The top 20 hub genes are the following: APP (amyloid beta A4 precursor protein), HSP90AA1 (heat shock protein 90 kDa alpha class A member 1), GRB2 (growth factor receptor-bound protein 2), SRC (v-src sarcoma viral oncogene homolog), TP53 (tumor protein p53), ESR1 (estrogen receptor 1), FN1 (fibronectin 1), TRAF6 (TNF receptor-associated factor 6), EGFR (epidermal growth factor receptor), SUMO1 (SMT3 suppressor of mif two 3 homolog 1), YWHAZ (14-3-3 zeta), MYC (v-myc myelocytomatosis viral oncogene homolog), CDK2 (cyclin-dependent kinase 2), HSPA8 (heat shock 70-kDa protein 8), MAPK1 (mitogen-activated protein kinase 1), AKT1 (v-akt murine thymoma viral oncogene homolog 1), COPS5 (COP9 constitutive photomorphogenic homolog subunit 5), MDM2 (Mdm2 p53 binding protein homolog), RELA (v-rel reticuloendotheliosis viral oncogene homolog A, NFKB3, p65), and HSPA4 (heat shock 70-kDa protein 4). APP is present in advanced human carotid plaques, in proximity to activated macrophages and platelets [23], and lack of APP attenuates atherogenesis and leads to plaque stability [24]. HSP90 is a candidate autoantigen, target of cellular and humoral immune reactions in patients with carotid atherosclerosis [25]. HSP90 expression is associated with features of plaque instability in advanced human lesions [26]. GRB2 is required for atherosclerotic lesion formation and uptake of oxidized LDL by macrophages [27]. In endothelial cell, SRC contributes to atherosclerotic lesion development by disrupting adherence junction integrity and promoting monocyte transmigration [28]. Increasing P53 activity protects against atherosclerosis by causing proliferation arrest of lesional macrophages [29]. The product of the MDM2 gene is a nuclear protein which forms a complex with P53, thereby inhibiting the negative regulatory effects of wild-type P53 on cell cycle progression. P53 and MDM2 are expressed in human atherosclerotic lesions; P53 and MDM2 may therefore play an important role in regulating cellularity and inflammatory activity in human atherosclerotic plaques [30, 31]. SUMOylation of P53 by SUMO1 contributes to the atherosclerotic plaque formation [32]. ESR1 is expressed in macrophages and other immune cells known to exert dramatic effects on glucose homeostasis. A study suggests that diminished ESR1 expression in hematopoietic/myeloid cells promotes aspects of the metabolic syndrome and accelerates atherosclerosis in female mice [33]. FN is one of the earliest extracellular matrix (ECM) proteins deposited at atherosclerosis-prone sites and was suggested to promote atherosclerotic lesion formation [34]. TRAF6 is expressed in atherosclerotic aortic tissue of low-density lipoprotein-null mice [35]. Endothelial-specific TRAF6 deficiency in females was associated with diminished atherosclerosis and decreased plaque macrophage burden [36]. EGFR mRNA was detected in atherosclerotic plaques but not in morphologically normal aortae and EGFR receptor staining co-localized with macrophage staining in these plaques [37]. Secreted from activated platelets, YWHAZ is present at the atherosclerotic plaques [38]. In cholesterol-fed roosters, MYC was seen in lipid-rich thickened intimal lesions of the entire aorta [39]. CDK2 negatively regulates neointimal thickening in animal models of restenosis and atherosclerosis, and its expression in human neointimal lesions is consistent with a protective role [40]. HSP70 (HSPA4 and HSPA8) is present in human atherosclerotic lesions [41]. Treatment with platelet-derived growth factor which caused vascular smooth muscle cell migration in an MAPK1 activation-dependent manner suggests a role for MAPK1 in the pathogenesis and/or progression of atherosclerosis [42]. AKT1 expression in vascular smooth muscle cells influences early and late stages of atherosclerosis. The absence of AKT1 in VSMCs induces features of plaque vulnerability including fibrous cap thinning and extensive necrotic core areas [43]. Macrophage migration inhibitory factor (MIF), a cytokine with potent inflammatory functions, was thus considered to be important in atherosclerotic lesion evolution. COPS5 is able to form complexes with MIF and serves critical regulatory functions in atherosclerotic lesion evolution [44]. The transcription factor NF-κB p65 is a key regulator in the regulation of an inflammatory response and in the pathology of atherosclerosis [45].

A limitation for text mining-based strategies is that there is no chance to discover new genes. In order to solve this problem, we enlarged the network by inducing new genes that are not reported to be involved in atherosclerosis. According to the rule of “guilty-by-association,” these new genes may be potential susceptibility genes. Finally, we made a list of 50 new genes, all of which have more than 44 connections with known genes (Additional file 4: Table S4).

## Conclusions

In summary, we have reported here the first systematic analysis of the molecular mechanism underlying atherosclerosis using a text mining approach. Our study provides a valuable resource for the in-depth understanding of the mechanism underlying atherosclerosis.

## Methods

### Identification of atherosclerosis-related genes by using text mining

The PubMed database was used as a source of literature for text mining. We conducted a search with the following combinations of query key words: “atherosclerosis” OR “atherogenesis” OR “atheroma” OR “atherosclerotic.” The search tag “[Title/Abstract]” was added after each keyword. The relevant articles were retrieved in XML format, which makes information extraction more precise due to the presence of content enclosed within XML tag pairs. For each article, titles and abstract texts were fetched using the dom4j XML parser class in JAVA. Abstract texts were further divided into sentences through sentence tokenizer implemented in LingPipe (Alias-I, Inc.). Text mining was performed at sentence level.

Gene mention recognition was performed using two different gene mention taggers, the hidden Markov model (HMM) tagger implemented in LingPipe and the ABNER tagger based on a machine learning system of conditional random fields (CRF) [46]. Gene mentions from both taggers were merged. Because researchers mention genes in a highly variable manner, we built a gene synonym dictionary from entrez gene database [47]. The dictionary was used for the gene name normalization process during which gene mentions were linked to entrez genes using exact string match. If multiple entrez genes share the same gene mention, the ambiguity was resolved manually. In order to minimize the false positive rate, we required the co-occurrence of atherosclerosis mention and gene mention within a single sentence. Finally, we compiled a complete list of atherosclerosis-related genes.

### Enrichment test of gene ontology (GO) terms

GO enrichment analysis was performed by using BiNGO 2.3 with GOslim dataset [48]. To test for enrichment, a hypergeometric test was conducted followed by Benjamini and Hochberg multiple test correction. The adjusted *p* value <0.01 was used as significance threshold to identify enriched categories.

### Pathway analysis

To rank overall importance of pathways involved in atherosclerosis, we calculated Fisher’s exact test *p* values and Benjamini-Hochberg adjusted *p* values through the DAVID bioinformatics resource 6.7 [49]. The significance threshold was set at 0.01. After enrichment tests, gene sets were collected for each pathway. Gene dependencies in a certain pathway were determined using the R package KEGGSOAP and visualized in Cytoscape [50, 51].

### Construction of protein-protein interaction (PPI) network

The genes associated with atherosclerosis were cross-referenced with the PINA2 database to create the PPI network [19]. The PINA2 database provides integrated and up-to-date protein-protein interactions available in IntAct, BioGRID, MINT, DIP, HPRD, and MIPS, which simplifies the task of inter-database mapping [8–13]. To query the PINA2 database, interaction was restricted to human and mouse and all kinds of experimental procedures were included. Cytoscape software was applied for visualization and analysis of PPI network. The topological parameters of PPI network were analyzed by NetworkAnalyzer [52]. The edges in the network were treated as undirected. The degree of a node was the number of its directly connecting neighbors in the network. The threshold degree value for hub genes was the mean plus two standard deviations. As a result, genes with a degree value of larger than 26 were considered hub genes. Hub genes and their connections were extracted from the whole network and rendered as a simplified sub-network.

## Additional files

Additional file 1: Table S1. A complete list of genes identified by text mining. (XLSX 148 kb) Additional file 2: Table S2. Gene ontology analysis. (XLSX 37 kb) Additional file 3: Table S3. Pathway enrichment analysis. (XLSX 14 kb) Additional file 4: Table S4. Potential susceptibility genes for atherosclerosis. (XLSX 12 kb)
